# Synthesis of Poly(butylene succinate) Catalyzed by Tetrabutyl Titanate and Supported by Activated Carbon

**DOI:** 10.3390/ma18061315

**Published:** 2025-03-17

**Authors:** Miao Chen, Guangxu Zhang, Ruolin Wang

**Affiliations:** School of Chemistry, Chemical Engineering and Life Sciences, Wuhan University of Technology, Wuhan 430070, China; 18071729194@163.com (M.C.); 282924@whut.edu.cn (R.W.)

**Keywords:** poly(butylene succinate), activated carbon, tetrabutyl titanate, catalytic synthesis

## Abstract

Polybutylene succinate (PBS) is a biodegradable aliphatic polyester with excellent thermal stability, mechanical properties, and processability. The synthesis of PBS typically employs titanium-based catalysts like tetrabutyl titanate (TBT) to accelerate the reaction. However, TBT acts as a homogeneous catalyst and is non-recyclable. This study aims to minimize the cost of recovering liquid TBT catalyst during PBS synthesis by using TBT-loaded activated carbon for direct esterification and optimizing the process conditions. The catalyst was analyzed using inductively coupled plasma emission spectroscopy, automated specific surface area and pore size analysis, X-ray diffraction, and Fourier-transform infrared spectroscopy. The product was evaluated through infrared spectroscopy, nuclear magnetic resonance hydrogen spectra, and gel permeation chromatography. The optimal process parameters were determined to be an esterification temperature of 170 °C, a polycondensation temperature of 235 °C, an acid-to-alcohol molar ratio of 1:1.2, a catalyst amount of 0.06 g, and a dehydration time of 3 h. Under these conditions, the weight-average molecular weight of PBS reached 47,655, reducing the catalyst usage from 0.5% to 0.3%, resulting in a 24.7% increase in catalytic efficiency compared to TBT, significantly lowering costs. After five cycles of reuse, the weight-average molecular weight of the product remained above 35,000. This study demonstrates that TBT-loaded activated carbon exhibits superior catalytic performance, offering a cost-effective and efficient method for industrial PBS production with broad application potential.

## 1. Introduction

Resource and environmental issues have become important constraints on the sustainable development of human society and the planet. Fully biodegradable materials, due to their green, environmentally friendly, and resource-saving characteristics, are gradually becoming a new emerging industry that leads technological innovation and economic development in the contemporary world [1,2,3]. The advancement of fully biodegradable materials holds substantial implications for mitigating carbon emissions and has consequently become a focal point in the global development of novel materials, representing a crucial frontier in international scientific and industrial competition [4,5].

Poly(butylene succinate) (PBS) began to enter the field of materials science research at the end of the 20th century [6,7]. With its excellent biocompatibility and outstanding biodegradability, it quickly became a focus of research on degradable polymer materials [8,9]. Its synthesis involves 1, 4-butanediol and succinic acid, both of which have well-established production processes in the petrochemical industry and biomass resource utilization [10,11]. In natural environments, PBS gradually degrades through enzymatic reactions into oligomers and small molecules, ultimately converting into carbon dioxide and water [12]. To reduce the environmental impacts of traditional petroleum-based packaging, biodegradable packaging has gradually become an ideal alternative to synthetic and petroleum-based packaging. Its rigid structure and highly transparent surface make it widely used in plastic bags, foodware, foam, and other fields. Additionally, it has broad application potential in agriculture, fisheries, packaging industries, automotive parts, electrical equipment, and aerospace industries [13,14,15,16].

The methods for synthesizing PBS mainly include direct esterification, transesterification, and chain extension. Direct esterification involves esterifying 1,4-butanediol with excess succinic acid at low temperatures, followed by polycondensation under high temperature and vacuum conditions to produce high-molecular-weight PBS polyester products. This method offers advantages such as a short reaction time, high product molecular weight, low equipment investment, and high process safety, making it suitable for industrial production [17,18]. However, high temperature and high vacuum conditions can lead to side reactions, causing the product to yellow and reducing its application value. Transesterification employs a catalyst to facilitate the ester exchange reaction between dimethyl succinate and a diol, removing methanol, and then conducting polycondensation to generate PBS. This method, however, incurs high raw material costs, requires long reaction times, and results in relatively lower-molecular-weight PBS. Chain extension begins with the formation of an initial polymer structure via the esterification reaction between succinic acid and 1,4-butanediol. Subsequently, chain extenders containing reactive groups are introduced. These reactive groups react with terminal hydroxyl groups to create ester bonds, thereby integrating the chain extender into the polymer chain ends and increasing the molecular weight. Common chain extenders include isocyanates, oxazolines, etc. [19,20]. However, in practical applications, this method reduces the biocompatibility and degradability of PBS. In China, direct esterification is predominantly used for PBS synthesis. During the synthesis of PBS via direct esterification, catalysts are commonly employed to accelerate the reaction rate. Typical catalysts include strong acid catalysts, such as toluene sulfonic acid, and titanium ester catalysts, such as tetrabutyl titanate [21].

Activated carbon (AC) is extensively utilized in catalysis owing to its substantial specific surface area, high mechanical strength, and rich presence of oxygen-containing functional groups, including carboxyl, ester, hydroxyl, phenolic, and quinone groups [22]. Activated carbon (AC) is widely used in catalysis due to its significant specific surface area, high mechanical strength, and abundant oxygen-containing functional groups such as carboxyl, ester, hydroxyl, phenolic, and quinone groups. Treatment with nitric acid can increase the number of oxygen-containing functional groups on the AC surface, enhancing the dispersion of active components and catalytic performance [23,24]. Titanium-based catalysts have become a research focus due to their high activity, non-pollution, and ability to provide polyester with higher brightness and transparency, as well as their affordable price, in the synthesis of copolymers [25]. Tetrabutyl titanate (TBT) is frequently used as a titanium catalyst in the PBS synthesis process, but the recovery of this liquid catalyst is difficult, leading to higher production costs [26,27,28].

This study explores the application of the impregnation technique to load tetrabutyl titanate onto activated carbon. Compared to liquid tetrabutyl titanate catalysts, the dosage is reduced from 0.05% [29] to 0.03% (based on the quality of succinic acid). Furthermore, the catalysts were recovered and recycled, and the results showed that the loaded catalysts had excellent catalytic activity and recyclability, which greatly reduced the production cost and was conducive to industrial application.

## 2. Materials and Methods

### 2.1. Materials

Nitric acid (HNO_3_), 1,4-butanediol, succinic acid, tetrabutyl titanate (TBT), triphenyl phosphite, and ethanol were purchased from Aladdin Chemical Reagent Co., Ltd., Shanghai, China. Activated carbon powder (AC) was purchased from China National Pharmaceutical Group Co., Ltd., Shanghai, China. The purity of the above reagents is analytical reagent.

### 2.2. Preparation of Catalysts

The catalysts were prepared using the classical impregnation method. The activated carbon was impregnated with 30% HNO_3_ at 20 °C for 5 h, filtered and dried at 80 °C until a constant was achieved. The resulting acidified activated carbon was designated as AC-HNO_3_. The TBT was dissolved in anhydrous ethanol to form an impregnation solution containing 15% tetrabutyl titanate by mass. Modified activated carbon, amounting to one-fifth of the impregnation solution’s mass, was then added. The impregnation process was conducted at 50 °C for 5 h and subsequently at 100 °C for 10 h. The mixture was washed three times with distilled water, filtered, and dried at 80 °C until a constant mass was achieved, resulting in the preparation of an activated carbon-loaded tetrabutyl titanate catalyst [30]. This catalyst was subsequently calcined under an argon atmosphere at 600 °C for 3 h to eliminate TBT. The resulting calcined catalyst was designated as AC-DS.

### 2.3. Synthesis of PBS

**Figure 1:**
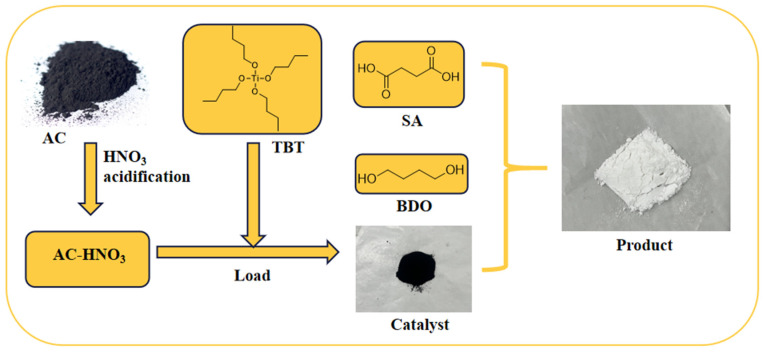
A schematic diagram illustrates the preparation of the catalyst and the synthesis process of PBS.

In a nitrogen atmosphere, 17.7 g (0.15 mol) of succinic acid, 16.2 g (0.18 mol) of 1,4-butanediol, and a specified quantity of AC-DS were introduced into a 500 mL four-neck flask fitted with a stirrer and a water separator. The mixture was heated to 165 °C and stirred for 3 h to promote esterification. Thereafter, 0.3 g of triphenyl phosphite was added as a thermal stabilizer, the temperature was raised to 230 °C, and the pressure was reduced to −0.1 MPa. Polycondensation was continued for an additional 3 h to obtain the crude product. The process flowchart is illustrated in Figure 1.

### 2.4. Purify

The crude product was dissolved in an appropriate amount of trichloromethane solution. After filtering out the catalyst, anhydrous methanol was added to the filtrate, resulting in the precipitation of polyester as white flocculent. The mixture was allowed to stand for a few minutes before filtration and subsequent washing with anhydrous methanol multiple times. The product was then dried under vacuum at 80 °C for use.

### 2.5. Catalyst Recycling

**Figure 2:**
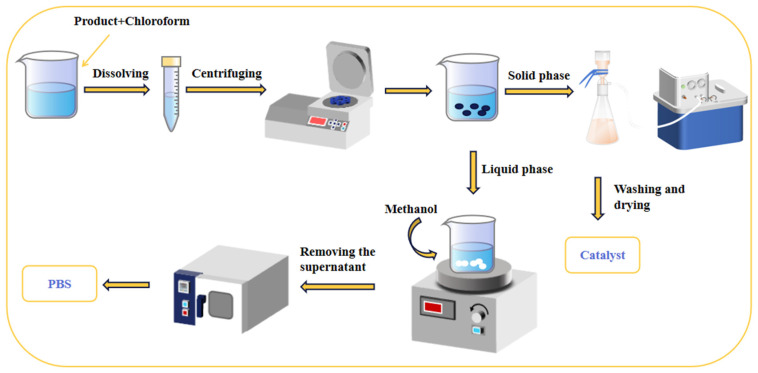
A schematic diagram illustrates the purification of the product and the recovery of the catalyst.

As depicted in Figure 2, the catalyst filtered out in Section 2.4 was washed several times with water. Subsequently, the resulting catalyst was dried under vacuum at 100 °C for future use.

### 2.6. Characterization Methods

The FT-IR spectra were obtained using a Nicolet6700 instrument (Thermo Fisher Scientific, Waltham, MA, USA) in reflection mode to analyze the chemical structure. The scanning range from 4000 cm^−1^ to 500 cm^−1^ was scanned 32 times. The M_w_ was determined by PL-GPC50 (Polymer Laboratories, Shropshire, UK). Chloroform served as the mobile phase, with a flow rate of 1.0 mL/min, a column temperature of 30 °C, and a sample solution concentration of 1.5 mg/mL. ^1^H NMR spectra were recorded using a Bruker 400 MHz nuclear magnetic resonance apparatus (Bruker, Bremen, Germany) at room temperature with tetramethylsilane as the internal standard and deuterated chloroform as the solvent. Titanium element was measured using a Prodigy-7 type plasma emission spectrometer (Teledyne Leeman Labs, Mason, OH, USA). Morphology and surface elemental distribution were examined by JSM-IT800 field-emission scanning electron microscopy (JEOL Ltd., Tokyo, Japan). Crystal phase structure analysis was conducted using an Empyrean-type X-ray diffractometer (Panaco, Amsterdam, The Netherlands), with a scanning range of 10~90° at a rate of 0.5°/min. Pore structure analysis was performed with an ASAP-2020 specific surface area and pore analyzer (Micromeritics, Norcross, GA, USA), employing the Brunauer–Emmett–Teller (BET) and Barrett–Jovner–Halenda (BJH) methods to calculate specific surface area and pore size. Intrinsic viscosity ([*η*], dL/g) was determined by preparing a PBS solution with a mass concentration of 0.5 g/dL in chloroform, measured at 25 °C using a Ubbelohde viscometer (Zhejing Glass Instrument Factory, Baoying County, Yangzhou, China) with an inner diameter of 0.3 to 0.4 mm [31], and [*η*] was calculated with Equations (1) and (2):

(1) $$\eta_{sp}=\frac{t-t_{0}}{t_{0}}$$

(2) $$[\eta ]=\frac{1}{C}\sqrt{2(\eta_{sp}-{ln\eta }_{r})}$$

where $\eta_{sp}$ is the specific viscosity of copolyester. *C* is the concentration of the solution, and t and $t_{0}$ are the flow time of the solution and pure solvent, respectively.

## 3. Results

### 3.1. Evaluation of Catalyst Activity

#### 3.1.1. Effect of Catalyst Loading Rate on Catalytic Performance

**Figure 3:**
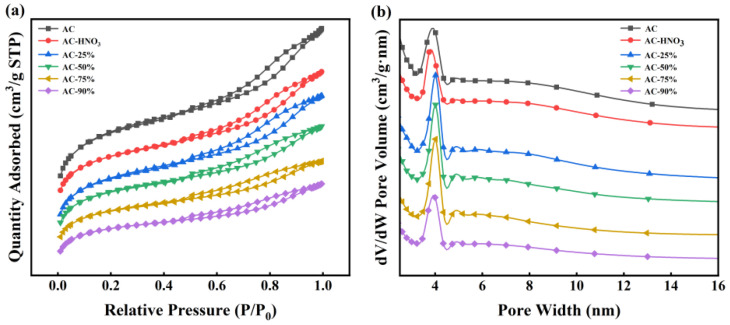
(**a**) N_2_ adsorption–desorption isotherms; (**b**) pore size distribution plots at different loading rates.

The activated carbon was acidified following the procedure detailed in Section 2.2. A series of new catalysts with loading rates of 25%, 50%, 75%, and 90% were prepared by impregnating TBT onto activated carbon, named AC-25%, AC-50%, AC-75%, and AC-90%, respectively. AC-HNO_3_, with a loading rate of 0%, served as the control. BET analysis was conducted to determine the specific surface area and pore structure of AC, AC-HNO_3_, AC-25%, AC-50%, AC-75%, and AC-90%, with results presented in Figure 3 and Table 1. Table 1 indicates that increasing the TBT loading rate slightly reduces the specific surface area, pore volume, and pore diameter due to TBT occupying some of the AC channels during the loading process. This reduction confirms successful TBT entry into the AC channels. Figure 3 reveals that all samples exhibit typical type IV isotherms with distinct hysteresis loops, indicating numerous mesoporous structures that are beneficial for substrate molecule entry into the catalyst. Additionally, the pore structure of the activated carbon remained largely unchanged post loading, suggesting that the carrier’s pore structure remains intact and unblocked during TBT loading. Compared to liquid TBT, the loaded catalyst demonstrates a larger pore volume and abundant mesoporous structures, facilitating better contact between the reactive substrate and active sites.

ICP analysis was conducted to ascertain the titanium content in AC-HNO_3_, AC-25%, AC-50%, AC-75%, and AC-90%. The findings are presented in Table 2. The reaction involved using 0.15 mol of succinic acid and 0.18 mol of 1,4-butanediol as starting materials, along with 0.12 g of catalyst at varying load rates. The dehydration step was performed at 165 °C for 3 h, as described in Section 2.3, followed by a vacuum reaction at 230 °C for 3 h. The viscosity and weight-average molecular weight (M_w_) of the products are also provided in Table 2.

According to Table 1 and Table 2, all the catalysts prepared have large specific surface areas and pore sizes, so the effect of the loading rate on the surface area and pore size is not particularly significant. However, both the intrinsic viscosity and the weight-average molecular weight of the product increase with the loading rate increasing. Without affecting the specific surface area and pore size, when the loading rate is 90%, the intrinsic viscosity and M_w_ of the product decrease significantly, which is not conducive to the progress of the polymerization reaction. Therefore, selecting a catalyst with a loading rate of 75% not only ensures that the catalyst has a large specific surface area and pore size but also ensures the amount of TBT, which is most conducive to the occurrence of a polymerization reaction.

#### 3.1.2. Evaluation of Activity of Different Catalysts

Experiments utilized 0.15 mol of succinic acid and 0.18 mol of 1,4-butanediol as starting materials, with varying amounts of TBT (0.03 g, 0.06 g, 0.09 g, 0.12 g, and 0.15 g). The reaction occurred at a dehydration temperature of 165 °C for 3 h and a condensation temperature of 230 °C under vacuum for another 3 h. Results are presented in Table 3. Initially, increasing TBT raised the product’s intrinsic viscosity and average molecular weight, but further additions beyond 0.12 g caused a decrease. Titanium content in AC-DS was determined via inductively coupled plasma spectroscopy, measuring 1.37%. Using tetrabutyl titanate as a catalyst, subsequent experiments included 0.12 g of AC-75%, 0.06 g of AC-HNO_3_, and 0.06 g of TBT (maintaining consistent titanium content), alongside a control group. These reactions used 0.1 mol of succinic acid and 0.12 mol of 1,4-butanediol at a dehydration temperature of 165 °C for 3 h and a condensation temperature of 230 °C under vacuum for 3 h. Results are also shown in Table 3.

Table 3 indicates that AC-75% significantly enhances product viscosity compared to no catalyst, liquid TBT, and TiO_2_ as catalysts. Using calcined AC-DS as a catalyst results in lower product viscosity than AC-75%. To further investigate the synergistic effect between the supported catalyst carrier and the active component, AC-HNO_3_ was physically mixed with TBT, maintaining the same titanium content for activity evaluation. Results confirmed that AC-75% remains the most effective catalyst. According to the preparation process of the catalysts in Section 2.2, it can be calculated that the TBT content in 0.12 g of AC-75% is approximately 0.05 g, indicating that 0.05 g of TBT in AC-75% provides superior catalytic performance than 0.12 g of liquid TBT. According to the catalytic mechanism of PBS synthesis [32], the original ligand on the central titanium atom of titanium-based catalysts undergoes a ligand exchange reaction with the hydroxyl end groups of monomers or prepolymers, forming active species within a specific timeframe. This timeframe correlates with the catalyst’s activity level [33]. Activated carbon’s surface contains numerous oxygen-containing functional groups, and the original ligand on the central atom Ti may partially exchange with ligands such as hydroxyl groups on activated carbon, thereby being activated to form active species, shortening the time required for the formation of active species during the reaction. Consequently, the catalyst demonstrates high activity.

### 3.2. Structural Characterization of Catalysts

#### 3.2.1. The FT-IR Spectra

**Figure 4:**
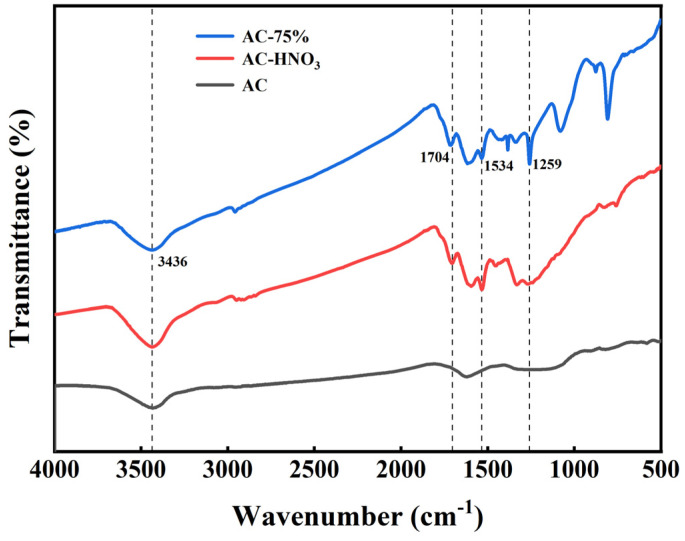
Infrared spectra of AC, AC-HNO_3,_ and AC-75%.

Infrared spectrum analyses of AC, AC-HNO_3_, and AC-75% were conducted, with results presented in Figure 4. At 1704 cm^−1^, the characteristic absorption peak of the carbonyl group is observed. In the range of 1350 to 1550 cm^−1^, the prominent absorption peak corresponds to the nitro group, while at 3436 cm^−1^, the absorption peak indicates the presence of the hydroxyl group. The adsorption of polar compounds is influenced by oxygen-containing groups on the surface of AC. Polar molecules with larger dipole moments are more affected by the properties and quantity of these oxygen-containing functional groups [34]. Acid treatment of AC enhances the presence of carbonyl, nitro, and hydroxyl groups, providing additional active sites for tetrabutyl titanate. Additionally, AC-75% exhibits a strong absorption peak at 1259 cm^−1^, within the infrared absorption range of the Ti-O bond (900 to 1300 cm^−1^), with the most pronounced peak at 1259 cm^−1^, corresponding to the highest electronic resonance frequency on the Ti-O bond. The results from ICP and infrared spectroscopy indicate that tetrabutyl titanate has been effectively loaded onto the activated carbon.

#### 3.2.2. SEM

**Figure 5:**
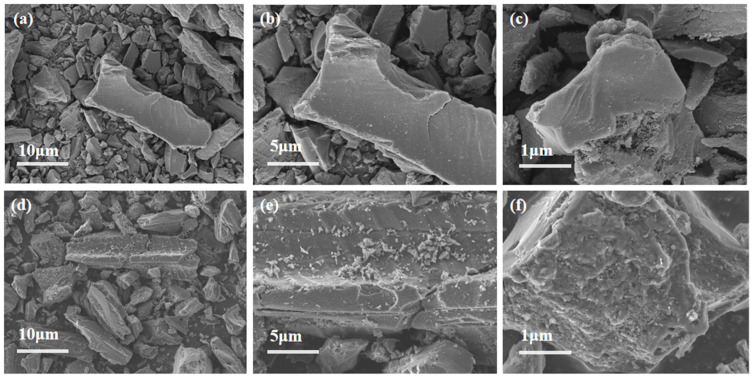
SEM image of (**a**–**c**) AC and (**d**–**f**) AC-75%.

**Figure 6:**
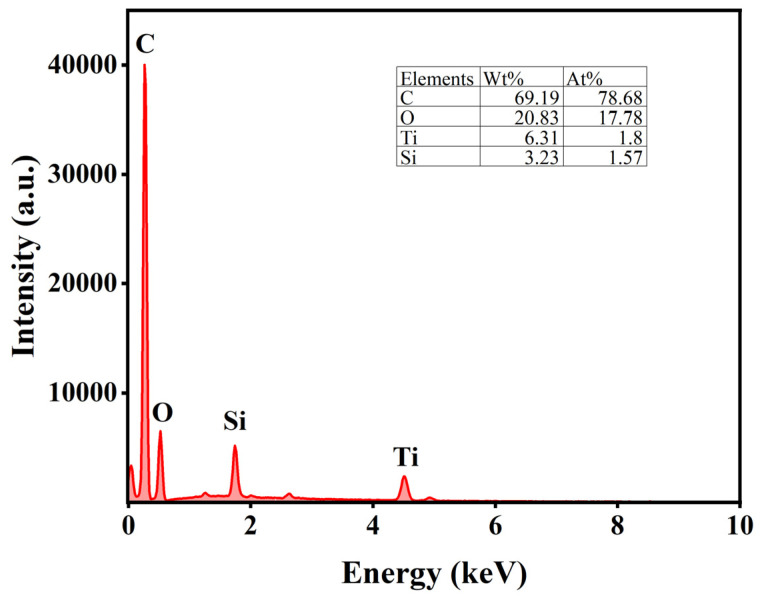
EDS spectrum of AC-75%.

The uniform distribution of active components significantly influences catalyst activity. Therefore, SEM was employed to analyze the morphology and properties of the catalysts. Figure 5a–c present the SEM results for AC, while Figure 5d–f display the SEM results for AC-75%. Additionally, the EDS analysis of AC-75% is shown in Figure 6. Observations from Figure 5 reveal numerous cracks and non-uniform pores on the AC surface, which can provide additional sites for TBT adhesion. A comparison of the SEM images of AC and AC-75% reveals that the AC surface is smooth, whereas AC-75% displays granular substances and cracks. The EDS spectrum of AC-75%, depicted in Figure 6, indicates the presence of C, O, Ti, and other elements, with Ti indicating successful TBT loading. Notably, despite TBT loading, no significant aggregation was observed on the activated carbon, while the dispersion of TBT within activated carbon is crucial for catalytic performance.

#### 3.2.3. XRD

**Figure 7:**
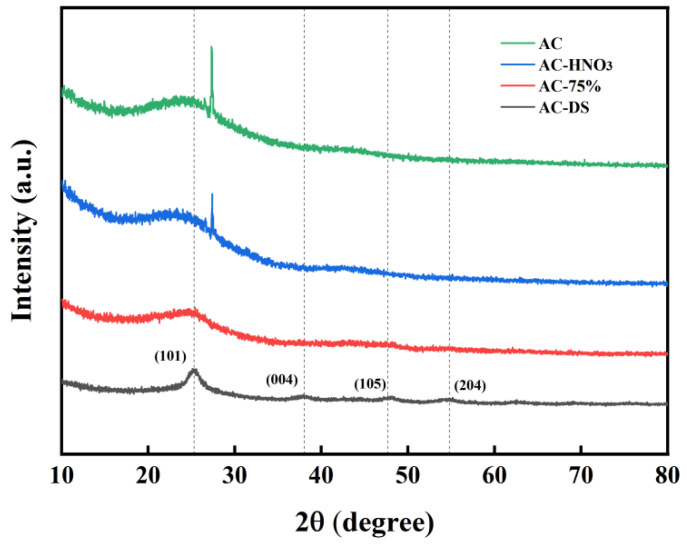
XRD patterns of AC, AC-HNO_3_, AC-75%, and AC-DS.

AC, AC-HNO_3_, AC-75%, and AC-DS were analyzed using X-ray diffraction. The XRD patterns of AC and AC-HNO_3_, shown in Figure 7, reveal that following HNO_3_ modification, the diffraction peak between 25° and 30° is weakened, suggesting damage to the crystal structure of AC. For AC-HNO_3_ and AC-75%, the diffraction peak between 25° and 30° significantly diminishes, rendering the microcrystalline structure of AC nearly undetectable. This reduction may be attributed to the elevated temperature of the active component TBT during loading compared to acidification, leading to the further acidification of some activated carbon. Consequently, the crystal phase of the carrier changes, causing severe lattice defects. After calcination, AC-DS exhibits four distinct diffraction peaks at 2θ = 25.3°, 38°, 47.7°, and 54.8°, corresponding to the (101), (004), (105), and (204) crystal planes of TiO_2_ [35], indicating that the remaining material is TiO_2_. In experiments involving a calcined catalyst (Table 3), the molecular weight is notably lower than that of AC-75%, providing strong evidence that the activation center is tetrabutyl titanate.

### 3.3. Optimization of Process Conditions for Catalytic Polymerization

#### 3.3.1. Dehydration Temperature

**Figure 8:**
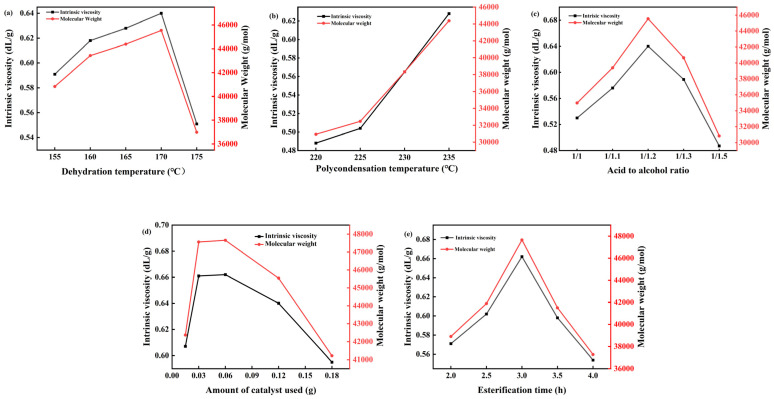
Curves of intrinsic viscosity and M_w_ of PBS with (**a**) Dehydration temperature, (**b**) Polycondensation temperature, (**c**) Acid-to-alcohol ratio, (**d**) Catalyst dosage, and (**e**) Esterification time.

The esterification stage involves the acid–alcohol dehydration reaction between 1,4-butanediol and succinic acid. This reversible reaction shifts towards the right when generated water is promptly removed. Increasing the esterification temperature expedites the discharge of water to the water separator. However, higher temperatures also accelerate side reactions, necessitating control within an optimal range [36]. Studies were conducted to determine the ideal reaction temperature, with results shown in Figure 8a. At a polycondensation temperature of 235 °C, an acid-to-alcohol ratio of 1:1.2, 0.12 g of catalyst, and an esterification time of 3 h, the product’s viscosity was examined at esterification temperatures of 155 °C, 160 °C, 165 °C, 170 °C, and 175 °C. The findings indicate that viscosity increases with a rising esterification temperature until it peaks at 170 °C, after which further increases lead to decreased viscosity. Consequently, 170 °C was chosen as the esterification temperature. Two replicate experiments were performed for each condition.

#### 3.3.2. Polycondensation Temperature

Oligomers formed through esterification reactions undergo polycondensation to form high polymers, with the polycondensation temperature significantly influencing the product’s molecular weight. This reaction necessitates elevated temperatures and a high vacuum environment. Increasing the temperature accelerates the reaction rate, while improving the vacuum facilitates the quicker removal of water produced during the reaction, thus promoting it. However, excessively high temperatures can cause polymer degradation, such as oxidative decomposition, resulting in issues like decreased product purity and discoloration, which limit its applications. To determine the optimal polymerization temperature, studies were conducted on the polycondensation temperature, with results depicted in Figure 8b. At an esterification temperature of 170 °C, an acid-to-alcohol ratio of 1:1.2, a catalyst dosage of 0.12 g, and an esterification time of 3 h, the product’s viscosity was examined at condensation temperatures of 220 °C, 225 °C, 230 °C, 235 °C, and 240 °C. Findings indicated that as the polycondensation temperature increased, so did the product’s viscosity. At 235 °C, the product exhibited maximum viscosity, while at 240 °C, the product became too viscous for further viscosity testing. Consequently, the polycondensation temperature was set at 235 °C.

#### 3.3.3. Acid–Alcohol Ratio

In the process of the experiment, with the continuous increase in temperature, side reactions continue to occur, and part of 1,4-butanediol will cyclize, so the amount of 1,4-butanediol needs to be slightly more than the amount of succinic acid to ensure the normal esterification reaction, but excessive 1,4-butanediol will cause a waste of raw materials, increasing cost. Vacuuming will also cause more load on the system, and the side reaction will also affect PBS’s purity and the molecular weight of PBS [37]. In the esterification process, based on the esterification temperature of 170 °C, the condensation temperature of 235 °C, and the amount of catalyst of 0.06 g, the esterification time was controlled for 3 h, and the viscosity of the product was investigated when the acid to alcohol ratio was 1:1, 1:1.1, 1:1.2, 1:1.3, and 1:1.5. The results are shown in Figure 8c, which shows that the viscosity of the product increases with the increase in the amount of 1,4-butanediol. When the acid-to-alcohol ratio is 1:1.2, the viscosity of the product is the highest, and then the acid-to-alcohol ratio is increased continuously, and the viscosity of the product decreases, probably because 1,4-butanediol is cycled into tetrahydrofuran. Therefore, the acid-to-alcohol ratio is 1:1.2.

#### 3.3.4. Catalyst Dosage

The addition of a catalyst will speed up the reaction speed and shorten the reaction time. If the amount of catalyst added is too small, the catalytic effect will not be obvious, but if the amount of catalyst added is too large, the reaction cost will increase, so the amount of catalyst needs to be explored. Based on the esterification temperature of 170 °C and the condensation temperature of 235 °C, the acid-to-alcohol ratio was controlled to 1:1.2, the esterification time was 3 h, and the viscosity of the product was investigated when the catalyst dosage was 0.015 g, 0.03 g, 0.06 g, 0.12 g, and 0.15 g, respectively. The viscosity of the product as a function of catalyst dosage was presented in Figure 8d. The experimental data demonstrated a non-monotonic relationship between catalyst dosage and product viscosity. Specifically, the viscosity reached its maximum value at an optimal catalyst dosage of 0.06 g. As the amount of catalyst increases, the product viscosity initially rises, reaching a peak at 0.06 g, after which it begins to decrease. Therefore, the optimal amount of catalyst is 0.06 g.

#### 3.3.5. Esterification Time

The duration of esterification plays a critical role in the synthesis process. Insufficient esterification time hinders the complete reaction between succinic acid and 1,4-butanediol, thereby impeding the formation of oligomers. Conversely, a prolonged esterification time promotes the cyclization of 1,4-butanediol into tetrahydrofuran, which is undesirable. Therefore, optimizing the esterification time is essential for achieving the desired product characteristics. In this study, the esterification process was conducted under the following conditions: an esterification temperature of 170 °C, a condensation temperature of 235 °C, an acid-to-alcohol molar ratio of 1:1.2, and a catalyst dosage of 0.06 g. The effect of esterification time on the product viscosity was investigated at intervals of 2 h, 2.5 h, 3 h, 3.5 h, and 4 h. The results, as depicted in Figure 8e, demonstrate that the viscosity of the product initially increased with prolonged esterification time, reaching a maximum at 3 h. Beyond this point, the further extension of the esterification time resulted in a decrease in product viscosity. Consequently, an optimal esterification time of 3 h was determined for this synthesis process.

#### 3.3.6. Service Life of Catalyst

**Figure 9:**
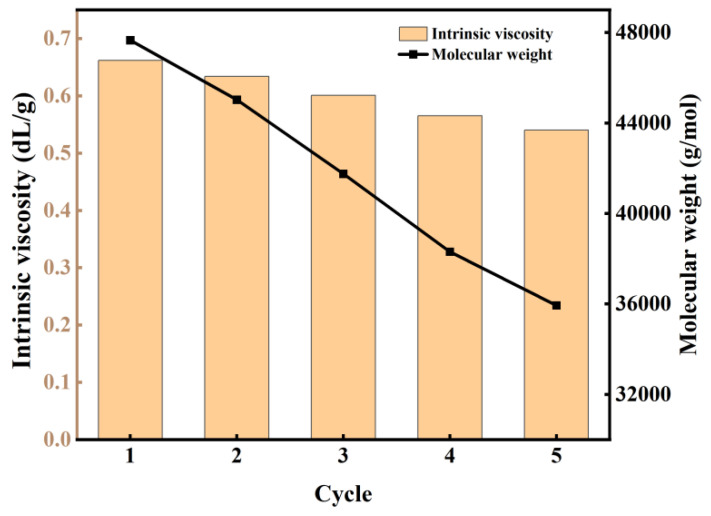
The change in intrinsic viscosity and M_w_ of PBS with the number of experiments.

To investigate the durability and reusability of the catalyst, five consecutive experimental runs were performed under identical reaction conditions. As depicted in Figure 9, a gradual decrease in the M_w_ of the produced polymer was observed with an increasing number of catalyst reuses. Notably, the catalyst maintained significant activity even after five cycles, consistently yielding products with M_w_ values exceeding 35,000 g/mol. This demonstrated stability in catalytic performance suggests promising potential for industrial applications, where catalyst longevity and consistent product quality are crucial parameters. The observed decline in M_w_ with repeated use may be attributed to partial deactivation or structural modifications of the catalyst, which warrants further investigation to optimize the catalyst’s lifetime and performance.

### 3.4. Structural Characterization of the Product

#### 3.4.1. Infrared Spectrum of the Product

**Figure 10:**
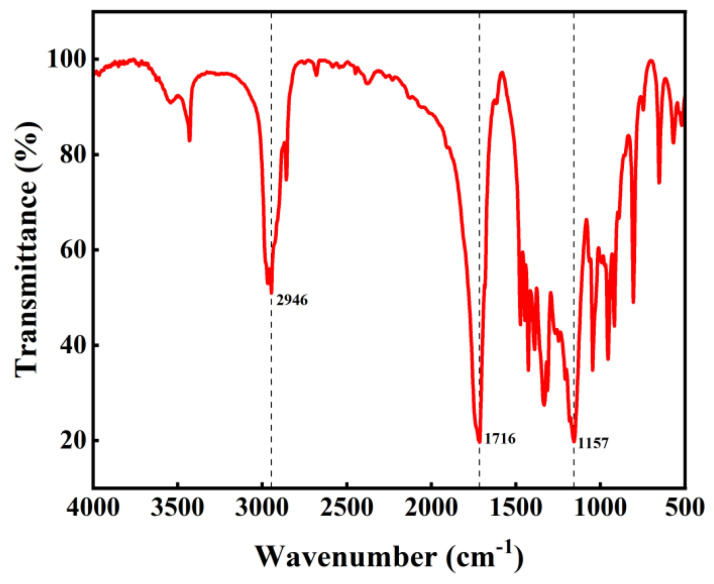
Infrared spectrum of product.

The infrared spectrum of the PBS product is shown in Figure 10. The absorption peak at 1716 cm^−1^ corresponds to the stretching vibration of the carbonyl group in the ester, while the peak at 1157 cm^−1^ represents the stretching vibration of the C-O single bond in the ester. These two distinct absorption peaks confirm the presence of ester groups [38]. The absorption peak at 2946 cm^−1^ corresponds to the stretching vibration of the methylene group.

#### 3.4.2. ^1^H NMR of the Product

**Figure 11:**
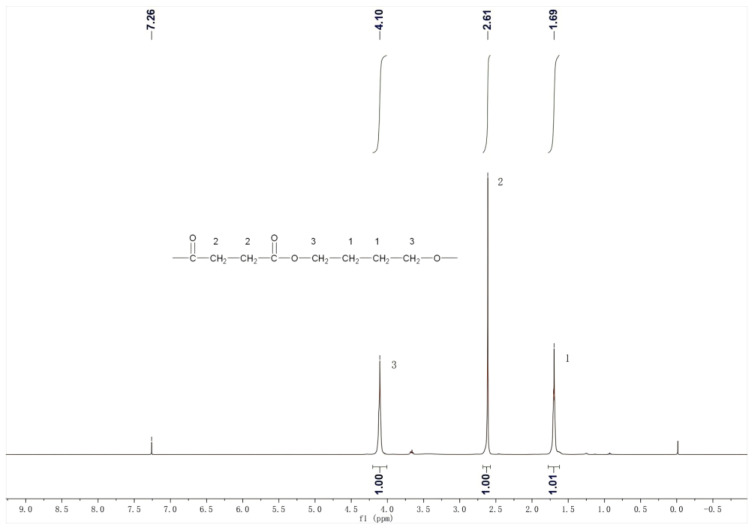
The ^1^H NMR character of the product.

The ^1^H NMR spectrum of PBS products is shown in Figure 11. In the 1,4-butanediol units within the polymer, the hydrogen atoms on the two carbons far from the ester group have a chemical shift of δ = 1.69, while the hydrogen atoms on the two carbons near the ester group have a chemical shift of δ = 4.10. In the succinic acid units, the hydrogen atoms on the two carbons have a chemical shift of δ = 2.61. According to integration, the area ratio of these three peaks is 1:1:1, corresponding to the ratio of three hydrogens in the PBS structural formula. Figure 11 is consistent with the standard spectrum of PBS. The ^1^H NMR spectrum of PBS products is depicted in Figure 11. For the 1,4-butanediol units within the polymer, the hydrogen atoms on the two carbons distant from the ester group exhibit a chemical shift of δ = 1.69, whereas those on the two carbons proximal to the ester group show a chemical shift of δ = 4.10. In the succinic acid units, the hydrogen atoms on the two carbons display a chemical shift of δ = 2.61. Integration reveals that the area ratio of these three peaks is 1:1:1, aligning with the ratio of three hydrogens in the PBS structural formula [39]. Figure 11 corresponds to the standard PBS spectrum.

Through the comprehensive analysis of the product’s structure using infrared spectroscopy and nuclear magnetic resonance hydrogen spectra, it was determined that the product is PBS.

## 4. Discussion

1. The TBT catalyst was prepared using an impregnation method. After acid treatment, the oxygen-containing functional groups on the AC surface increased, providing more active sites for the supported TBT. The original ligands on the central Ti atom may partially exchange with ligands such as hydroxyl groups on the activated carbon, thus being activated to form active species, which shortens the time required for active species formation during the reaction. Therefore, this catalyst exhibits high activity.
2. The experimental determination of the optimal process conditions for synthesizing PBS revealed that the ideal catalyst loading is 75%, the esterification temperature is 170 °C, the polycondensation temperature is 235 °C, with a molar ratio of succinic acid: 1,4-butanediol of 1:1.2, catalyst usage of 0.06 g, dehydration time of 3 h, and optimal catalyst amount of 0.3% of succinic acid mass. The resulting product has a number-average molecular weight of 47,655. Compared to existing processes, this study’s findings can significantly reduce the production cost of PBS.
3. After being reused five times, the product’s weight-average molecular weight still exceeded 35,000, indicating that the catalyst supported on activated carbon has excellent catalytic performance and a long service life, with good application prospects. However, this study also has certain limitations; for example, after the catalyst was cycled five times, the product’s molecular weight decreased by 24% compared to its initial use, suggesting that the stability of the catalyst needs further improvement.

## 5. Conclusions

This study presents a novel method for the catalytic synthesis of PBS, involving the transformation of homogeneous catalysts into heterogeneous catalysts. Due to the presence of oxygen-containing functional groups on the surface of activated carbon, active species easily form, resulting in high catalyst activity. Compared to liquid catalysts, supported catalysts have larger pore volumes and abundant mesoporous structures, which facilitate contact between reaction substrates and active sites. Experimental results show that when using the AC-75% catalyst to synthesize PBS, the catalyst amount was reduced from 0.5% to 0.3%, with a catalytic effect 24.7% higher than TBT. The AC-75% catalyst exhibits excellent catalytic performance and recyclability, making it cost effective for industrial production.

## Figures and Tables

**Table 1:** Specific surface area, pore volume, and pore size of catalysts with different loading rates.

Catalyst	Specific Surface Area (m^2^/g)	Pore Volume (cm^3^/g)	Pore Diameter (nm)
AC	1551.9551	1.2772	4.2723
AC-HNO_3_	1374.9072	1.1023	4.2541
AC-25%	1176.6737	0.9141	4.2160
AC-50%	1023.7634	0.8627	4.0830
AC-75%	1007.6578	0.8041	4.0376
AC-90%	997.9885	0.7443	3.8842

**Table 2:** The viscosity and weight-average molecular weight of the products obtained using catalysts with different loading rates.

Catalyst	Titanium Content	Intrinsic Viscosity (dL/g)	M_w_ × 10^−4^ (g/mol)
AC-HNO_3_	0.02%	0.372	1.9778
AC-25%	3.07%	0.461	2.8334
AC-50%	5.03%	0.487	3.0833
AC-75%	7.18%	0.551	3.6985
AC-90%	8.95%	0.412	2.3624

**Table 3:** The viscosity and weight-average molecular weight of the products obtained with different catalysts.

Catalyst	Catalyst Dosage (g)	Titanium Content (g)	Intrinsic Viscosity (dL/g)	M_w_ × 10^−4^ (g/mol)
Catalyst-free	0	0	0.358	1.8432
TBT	0.03	0.0042	0.403	2.2758
TBT	0.06	0.0084	0.467	2.4681
TBT	0.09	0.0127	0.518	3.3813
TBT	0.12	0.0169	0.453	2.7564
TBT	0.15	0.0211	0.437	2.6026
TiO_2_	0.09	0.0540	0.372	1.9778
AC-75%	0.09	0.0065	0.578	3.9580
AC-DS	0.47	0.0065	0.377	2.0259
0.04 gAC-HNO_3_ + 0.05 gTBT	0.09	0.0065	0.412	2.3623

## Data Availability

The original contributions presented in this study are included in the article. Further inquiries can be directed to the corresponding author.
